# Right atrial thrombus in a neonate with Aspergillus colonization secondary to central venous catheter: a case report

**DOI:** 10.1093/ehjcr/ytaf049

**Published:** 2025-02-03

**Authors:** Hassan El-Shirbiny, Mohamed Ramadan, Mahmoud Gomaa

**Affiliations:** Cardiology Department, Kafrelsheikh University Hospital, El-Geish Street, Kafrelsheikh 33516, Egypt; Cardiology Department, Kafrelsheikh University Faculty of Medicine, Kafrelsheikh 33516, Egypt; Cardiology Department, Kafrelsheikh University Hospital, El-Geish Street, Kafrelsheikh 33516, Egypt; Cardiology Department, Kafrelsheikh University Hospital, El-Geish Street, Kafrelsheikh 33516, Egypt

**Keywords:** Central venous catheters, Atrial thrombosis, Cardiac mass, Neonatal echocardiography, Neonatal sepsis, Aspergillosis, Case report

## Abstract

**Background:**

Central venous catheter-related thrombosis (CRT) is a recognized complication, particularly in patients with underlying hypercoagulable states. This condition can precipitate several complications, including secondary infections, necessitating immediate and effective diagnosis and management.

**Case summary:**

In this report, we present a case of a 13-day-old neonate admitted to the neonatal intensive care unit with severe respiratory distress, necessitating the insertion of a central venous catheter for optimal nutrition and treatment. Persistent fever and worsening general condition prompted further investigations, including echocardiography, which revealed a sizeable right atrial mass. Surgical removal of the mass was performed, and subsequent analysis identified thrombosis complicated by Aspergillus colonization. Postoperative management included anticoagulation and antifungal therapy, which were continued for 3 weeks until the patient’s condition improved, leading to discharge.

**Discussion:**

This case highlights the importance of accurate differential diagnosis of cardiac masses, early recognition and management of CRT in neonates, and the need for timely treatment of opportunistic infections like invasive Aspergillosis for optimal outcomes.

**Learning points**

- Vascular procedures demand strict aseptic techniques, but infection risks persist, particularly in vulnerable groups such as preterm infants.
- Accurate differential diagnosis of cardiac thrombi and other similar masses using comprehensive clinical evaluation and imaging techniques should be thoroughly studied. Although these might not always be feasible, especially in centres with limited resources, they can hugely improve the outcome by earlier diagnosis and subsequent intervention, and shorter hospital stay.
- It is important to consider how preterm neonates are prone to opportunistic infections such as invasive Aspergillosis and how timely diagnosis and management are critical for desired outcomes.

## Introduction

In preterm neonates, central venous catheters (CVCs) are commonly used for the administration of medications or parenteral nutrition. However, a significant risk associated with these catheters is the development of thrombotic complications.^1^ These thrombi may occasionally resolve spontaneously but can also result in severe complications such as secondary infection.^2^ In this report, we present a case of a preterm neonate who developed a large intra-atrial thrombus that was surgically removed and histopathological analysis showed that it had been further complicated by Aspergillus colonization, a rare and potentially fatal complication.

## Summary figure

**Figure:**
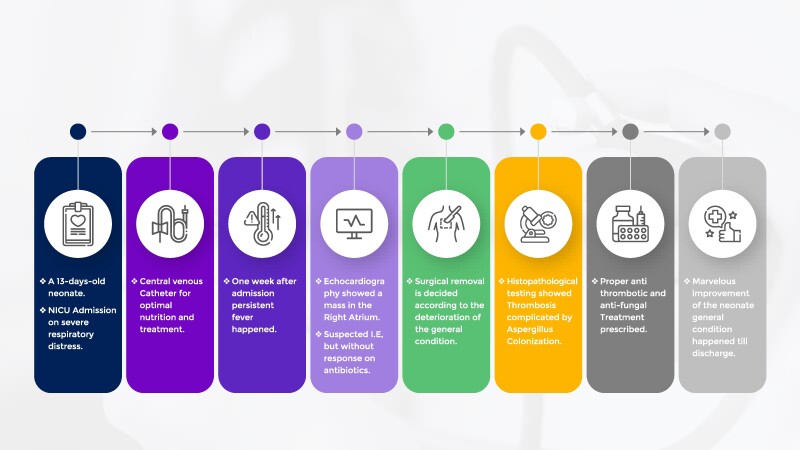


## Case presentation

This is a case of a 13-day-old male neonate who was born preterm at 36 weeks via caesarean section, with a birth weight of 1800 g. He was admitted to the neonatal intensive care unit (NICU) due to his parents’ concerns about symptoms suggestive of respiratory distress. They reported poor oral intake and a recent 100 g loss of weight and observed rapid breathing and occasional bluish discolouration. His mother, a 35-year-old G2P2, pre-diabetic, with a body mass index of 38 kg/m^2^ and a history of deep venous thrombosis after her first delivery. Unfortunately, she missed most of her prenatal visits. The pregnancy was normal but occurred only one year after the first delivery. The caesarean section was uncomplicated.

Upon physical examination, the patient exhibited tachypnoea, tachycardia, significant intercostal retractions, and nasal flaring, accompanied by intermittent expiratory grunting. The skin was pale with perioral cyanosis. Auscultation revealed bilateral diminished breath sounds with crackles.

He was lethargic, hypotonic with decreased responsiveness, and developed fever (38.3 °C) 48 h after birth. Additionally, he had reduced oral intake and signs of dehydration, including dry mucous membranes and a sunken fontanelle.

**Figure 1:**
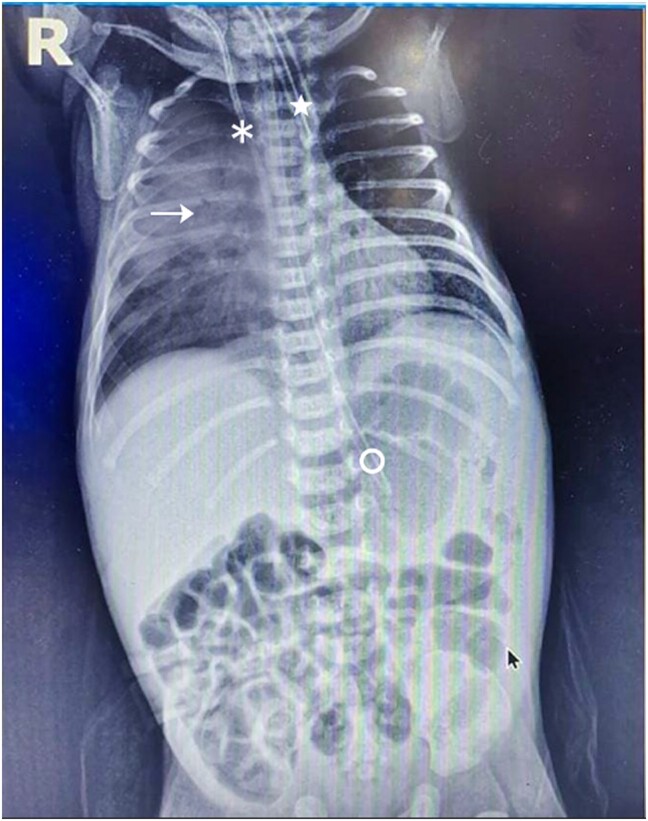
Chest X-ray showing right central venous catheter (asterisk), endotracheal tube (star), nasogastric tube (circle) and right lung consolidation (arrow).

On admission, the patient received supplemental oxygen and was closely monitored for respiratory status, with mechanical ventilation considered if needed. Initial diagnostics included a complete blood count (with a total leukocyte count of 12 000), blood cultures, and arterial blood gases (ABG), which showed respiratory alkalosis. Due to difficulty with peripheral access, a central venous line was inserted for reliable administration of IV fluids and medications. An X-ray indicated transient tachypnoea of the newborn, showing hyperinflation and prominent vascular markings along with right lung mid-infiltrates suggesting pneumonia. The central line appeared properly placed without complications (*Figure 1*). Antipyretics and broad-spectrum antibiotics (ampicillin 100 mg/kg IV every 12 h and gentamicin 4 mg/kg IV every 24 h) were started within the first hour after pan-cultures had been withdrawn. Over the next 24 h, the patient’s tachypnoea and ABGs showed slight improvement. Supportive care, including parenteral nutrition, was provided throughout the first week.

**Figure 2:**
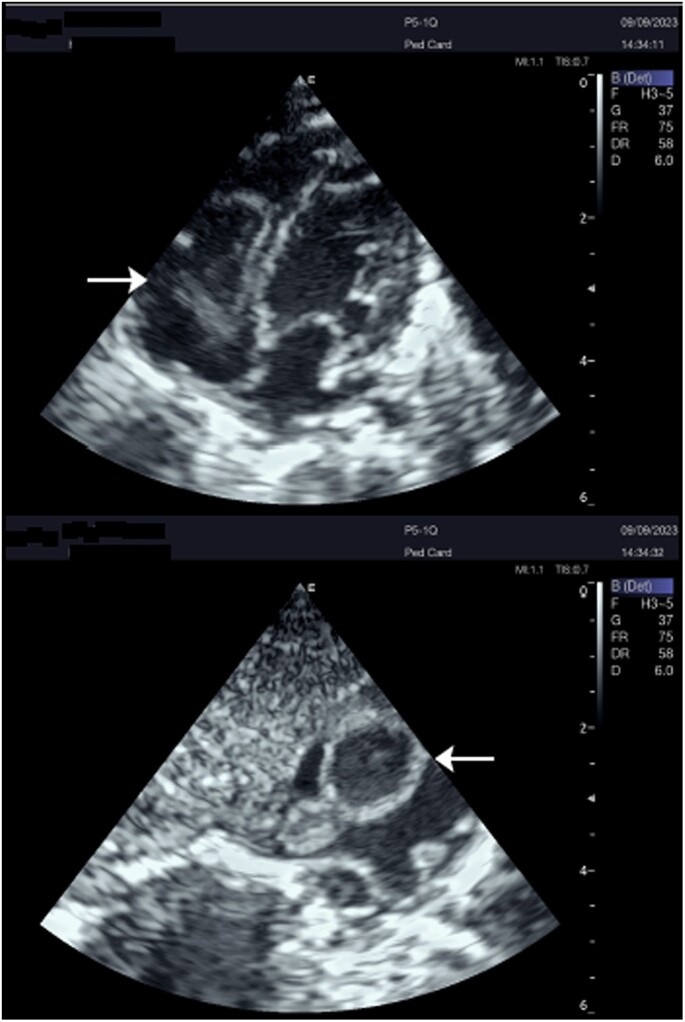
A pedunculated mass in the right atrium (arrow).

**Figure 3:**
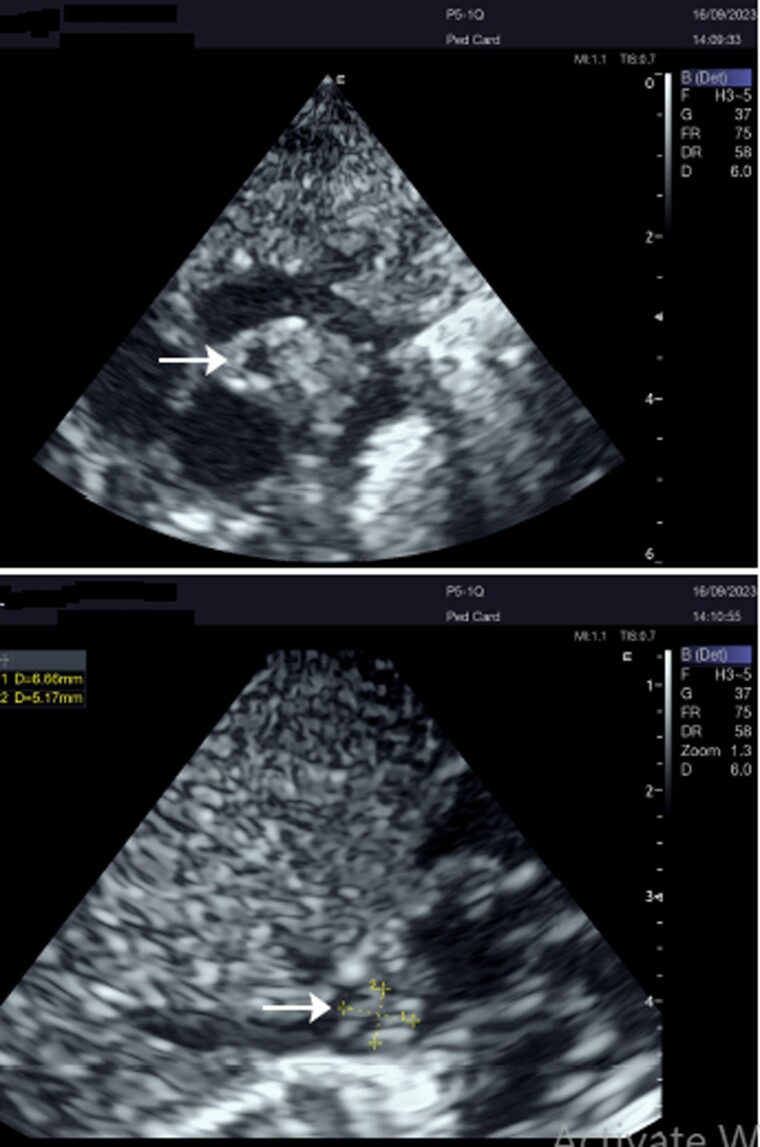
A large pedunculated mass seems to have a pedicle originating from the inferior vena cava (arrow).

Over the next 5 days, the fever resolved, and the patient’s respiratory status improved. However, after a week from admission, mid-diastolic plop was auscultated, and the fever reoccurred. Blood cultures were repeated, and a transthoracic echocardiogram (TTE) was performed, revealing an atrial pedunculated mass partially obstructing the tricuspid valve (TV) with a peak transvalvular gradient of 15 mmHg and a right atrial pressure of 11 mmHg (*Figure 2*). A diagnosis of infective endocarditis was considered, and the central venous line was identified as the likely source of infection. Despite aggressive antibiotic therapy with vancomycin (15 mg/kg QID) and gentamycin (3 mg/kg once daily), the fever persisted, and the patient’s condition has worsened. Repeated blood culture results were negative for aerobic and anaerobic bacteria, and follow-up TTE showed an increase in the size of the right atrial pedunculated mass with diastolic extension to the inferior vena cava (IVC) where the pedicle seems to be attached to (*Figure 3*).

**Figure 4:**
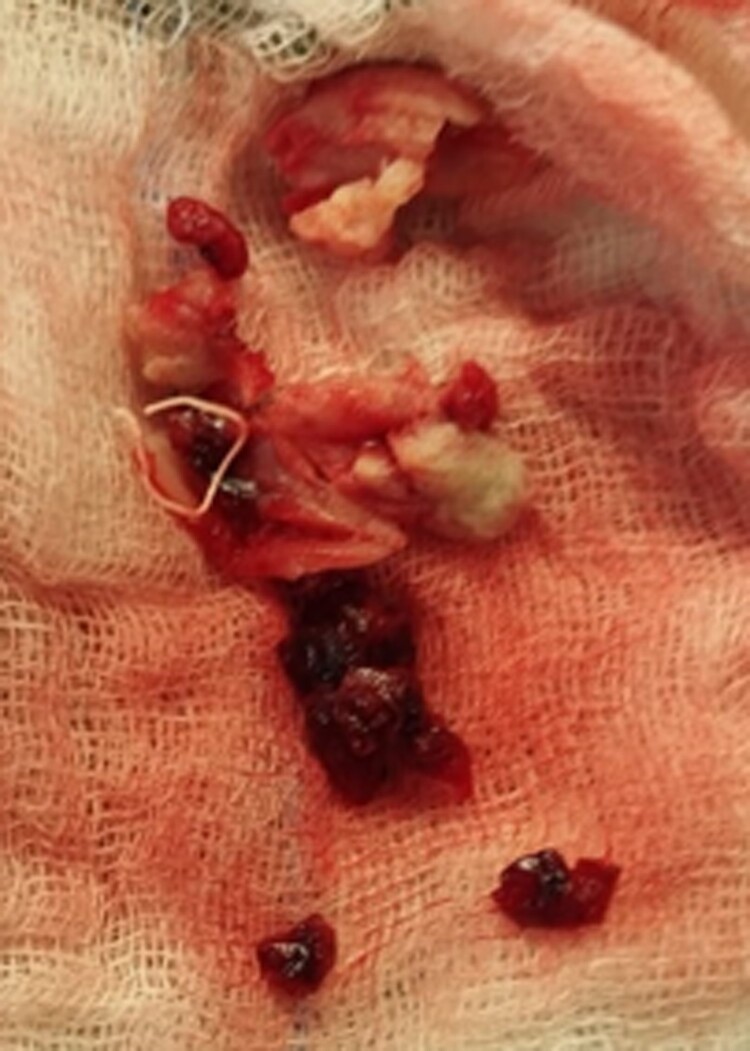
Surgical removal of the right atrial mass.

Therefore, the decision was made to proceed with open-heart surgery for thrombectomy. During the procedure, the large thrombus was removed from the right atrium (*Figure 4*). Histopathological analysis of the thrombus revealed fungal colonization by Aspergillus, a fungus commonly colonizing in tissue masses and often missed in fungal blood cultures.^3^

Postoperatively, the central venous line was replaced. Antibiotics were stopped and the patient was started on anticoagulants (SC enoxaparin 1.5 mg/kg BID) and antifungal therapy (IV voriconazole loading with 6 mg/kg BID in the first 24 h and maintenance with 4 mg/kg BID thereafter). Over the following 3 weeks, the patient showed significant clinical improvement, with resolution of the fever and stabilization of respiratory and cardiac function. The patient was discharged from the NICU after stopping the enoxaparin and with a plan for continuing oral voriconazole for an additional 4 weeks with regular follow-up of liver and kidney function tests, which were normal. Follow-up TTEs confirmed no recurrence of the thrombus.

## Discussion

This case report highlights a rare complication of CVC-related thrombosis (CRT) that was managed appropriately. Thrombosis can occur in patients with CVCs since they affect all three components of Virchow’s triad: hypercoagulability, endothelial injury, and stasis.^4^ Central venous catheter-related thrombosis should be considered, especially since the incidence of CRT is estimated to range from 0.4 to 1.0 per 10 000 individuals.^5^ Patients with hypercoagulable status, such as sepsis-induced coagulopathy, which was the case with our patient, are more prone to CRT.

Being immunocompromised, preterm neonates are at risk for developing rare opportunistic infections such as invasive Aspergillosis.^6^ The review by Weimer *et al*.^7^ highlighted that those invasive fungal infections, primarily caused by Candida and Aspergillus species, are major contributors to morbidity and mortality in neonates, particularly those who are preterm or have very low birth weight.

Here, our case has developed a large atrial thrombus that expanded to reach the IVC and caused partial TV obstruction as well. Initially, it was mistakenly managed as a vegetation of bacterial infective endocarditis. However, analysis after surgical removal showed Aspergillus colonization and antifungal therapy was initiated and successfully managed the patient's condition.

Accurate differential diagnosis of cardiac masses is essential for effective clinical management. This process involves integrating patient history, clinical examination, and cardiovascular imaging data.^8^ Initial diagnoses are often made using echocardiography. However, the findings may not be specific. The study by Scheffel *et al*.^9^ explored how cardiac computed tomography (CT) imaging can distinguish atrial thrombi from myxomas and other types of masses. Unfortunately, cardiac CT was not feasible with our patient due to resource constraints.

In the literature, a notable study explored the pro-thrombotic effects of fungal infection, particularly Aspergillus, and concluded that angioinvasion and thrombosis by Aspergillus hyphae *in vivo* may result from endothelial cell invasion, injury induction, and tissue factor activity stimulation.^10^ There are only a few comparable case reports, for example, Gover *et al*.^11^ described a case involving a preterm infant with a catheter-related right atrial thrombus, which was successfully managed with aspiration thrombectomy. Treatment options for right heart thrombi include anticoagulation, systemic thrombolysis, and surgical thrombectomy. The European Cooperative Study reported mortality rates of 60% for anticoagulation, 40% for thrombolysis, and 27% for surgery, suggesting surgery may be most effective.^12^ In another case, a 19-year-old patient with a large, highly mobile right atrial mass underwent urgent cardiac surgery, as recommended by the heart team, who deemed it the most appropriate management option.^13^

In conclusion, this case report highlights the importance of recognizing and managing CRT in neonates, especially when sepsis-induced coagulopathy may be present. Additionally, accurate differential diagnosis of cardiac thrombi and other similar masses using comprehensive clinical evaluation and imaging techniques should be thoroughly studied. Finally, it is important to consider how preterm neonates are prone to opportunistic infections such as invasive Aspergillosis and how timely diagnosis and management are critical for desired outcomes.

## Supplementary Material

ytaf049_Supplementary_Data

## Data Availability

All the data underlying this article are available in its online Supplementary material.

## Lead author biography

As an aspiring cardiologist, I hold a profound passion for cardiology. My academic training and hands-on clinical experiences have equipped me with a solid understanding of cardiovascular physiology, diagnostic methods, and advanced treatment options. I am dedicated to delivering exceptional, patient-centred care, merging my clinical skills with a compassionate approach. My goal is to significantly impact the lives of individuals affected by cardiac conditions, helping them achieve optimal heart health and enhancing their quality of life. Additionally, I have a keen interest in scientific research and am eager to contribute to the advancement of medical knowledge and the development of innovative solutions within the field of cardiology.

## Notes

**Consent:** The authors confirm that all materials, including a written consent from the parents for submission and publication of this case report, in the form of images and associated text, have been obtained from the patient, in the same line with COPE guidance.

**Ethics approval and consent to participate:** A written consent was obtained from the parents of the newborn for documentation and publication of the data for educational purposes. MKSU 52-10-12.

**Funding:** The authors have no budget or financial relationships to disclose.
